# Evaluation of Morphological, Phytochemical, and Antioxidant Properties of Wild *Equisetum arvense*: Impact of Aquaponic Cultivation

**DOI:** 10.1155/ianc/2934379

**Published:** 2026-10-11

**Authors:** Salman Ranjbar, Alireza Ladan Moghadam, Shahroz Habibi Kutani, Mehran Mahdipour, Mehdi Maham

**Affiliations:** ^1^ Department of Horticultural Sciences AK.C. Islamic Azad University Aliabad Katoul Iran azad.ac.ir; ^2^ Department of Horticulture Sciences Ga.C. Islamic Azad University Garmsar Iran azad.ac.ir; ^3^ Department of Agriculture Go.C. Islamic Azad University Gorgan Iran azad.ac.ir; ^4^ Department of Veterinary Ga. C. Islamic Azad University Garmsar Iran azad.ac.ir; ^5^ Department of Chemistry AK.C. Islamic Azad University Aliabad Katoul Iran azad.ac.ir

**Keywords:** antioxidant activity, aquaponic cultivation, *Equisetum arvense*, phytochemical compounds

## Abstract

*Equisetum arvense* is known as a rich source of phytochemicals, including flavonoids and phenolic acids, in traditional and modern medicine. This study aimed to compare morphological characteristics, phytochemical composition, and antioxidant activity of wild versus aquaponically cultivated *E*. *arvense* to assess the impact of cultivation method on plant quality. Plant samples were collected from wild areas and an aquaponic cultivation system. Morphological characteristics were measured. Active compounds were analyzed using HPLC, as well as Folin–Ciocalteu and aluminum chloride methods to measure the concentrations of phenolic compounds and flavonoids. The antioxidant activity of the extracts was also evaluated using DPPH tests. Data were analyzed using an independent *t*‐test. Morphological comparison showed that aquaponic plants had significantly greater height, stem diameter, node spacing, and internode spacing, while wild plants had more leaves per node (*p* < 0.05 to *p* < 0.01). No significant differences were observed for internodes per leaf and sheath teeth(ns). HPLC analysis revealed that wild extracts contained higher concentrations of chlorogenic acid (58 mg/g), ferulic acid (11.83 mg/g), and rutin (10.98 mg/g), while aquaponic samples were richer in rosmarinic acid (7.34 mg/g). Total phenolic and flavonoid contents were higher in wild plants (55.4 mg GAE/g and 31.25 mg RE/g) compared to aquaponic ones (45.4 mg GAE/g and 21.25 mg RE/g). Antioxidant activity (IC50) was 282.83 μg/mL for wild and 182.83 μg/mL for aquaponic samples. These results suggest that cultivation method critically determines the chemical composition and biological activity of *E*. *arvense*. Despite lower phenolic levels, aquaponic plants showed superior antioxidant activity. Each method offers distinct advantages, and neither is absolutely superior. Aquaponic systems may serve as a sustainable platform for producing medicinal plant extracts with potential therapeutic value.

## 1. Introduction

Medicinal plants have long been used in traditional medicine in various countries, including Iran, to treat diseases. Iran, with its diverse climate and rich vegetation, hosts a variety of such plants that have been cultivated and exploited since ancient times [1]. *E*. *arvense*, belonging to the Equisetaceae family and commonly known as horsetail, is a plant species with a long history of use in traditional medicine and as a source of silica [2]. This plant is traditionally collected from nature in certain areas for food and medicinal purposes, suggesting that it may be abundant and available in its natural habitat [3]. The phytochemical compounds of this plant, including flavonoids, phenolic acids, and saponins, have been associated with various potential health benefits, such as anti‐inflammatory and antioxidant effects [4]. Cultivation methods, as one of the main factors in improving plant growth, have a significant impact on various plant characteristics, including morphological, phytochemical, and bioactive properties. One of the modern cultivation methods is aquaponics, which combines aquaculture and hydroponic cultivation systems. In this system, it is possible to produce aquatic animals and plants simultaneously, optimizing the use of water resources and reducing the need for chemical fertilizers. This method has been proposed as a sustainable model for agriculture, especially in water‐scarce regions, and can have positive effects on the phytochemical and bioactive properties of plants [5, 6].

Research has shown that there is high variability in the morphological and phytochemical characteristics of *E*. *arvense*, especially in terms of total phenolic content (TPC) and antioxidant activity. This variability can be attributed to various factors, including environmental conditions and cultivation methods. One study showed that different ecotypes of *E. arvense* differed in height, width, stem dry weight, and silica content [7]. Another study identified various phenolic compounds, including gallic acid and isoquercitrin, that could influence their medicinal properties [8]. Additionally, a study found that the silicon content of *E. arvense* can vary, which could be related to its cultivation and use [9]. A study comparing the yield, nutrient uptake, and water use efficiency of *Nasturtium officinale* grown in aquaponic, hydroponic, and soil systems showed that soilless systems resulted in faster plant growth and higher yields [10]. Another study demonstrated that aquaponic cultivation of lemon basil, by feeding fish with 3% yeast biomass, resulted in a significant increase in polyphenol content and antioxidant activity compared to wild plants. These findings highlight the great potential of aquaponics in optimizing biologically active compounds [11]. Furthermore, a study showed that aquaponic cultivation of vegetables such as red pepper and tomato in the summer resulted in significant increases in IC50 concentrations, antioxidant capacity, and the content of phenolic and flavonoid compounds compared to wild plants. These findings suggest the significant potential of aquaponics in optimizing the biologically active compounds of plants [12]. The results of a study evaluating hydroponics and aquaponics indicated that an aquaponic cultivation system for strawberries (*Primoris*) can effectively maintain fruit production and quality while reducing the need for chemical fertilizers. These results clearly demonstrate the potential of aquaponics as a more sustainable option in modern agriculture [13].

In summary, the study of the morphological, phytochemical, and antioxidant properties of medicinal plants cultivated in different systems compared to their wild type provides valuable information about the effect of these cultivation systems, especially aquaponics, on the chemical compounds present in the plants and their biological properties. In recent years, due to the increasing demand for medicinal plants and the need for sustainable and environmentally friendly production, alongside the numerous advantages of aquaponic cultivation, including resource optimization and the production of quality products, this type of cultivation has attracted the attention of researchers for various plants, resulting in numerous studies in this field [9–13]. However, the effect of aquaponic cultivation on the morphological, phytochemical, and antioxidant properties of *E*. *arvense* remains unknown, and reliable evidence and reports in this area are very limited. The results of existing studies have also not been adequately presented. Thus, this study addresses a clear knowledge gap. Accordingly, given that the type of cultivation can have a significant effect on the morphological characteristics and various properties of the plant extract, this study aims to compare wild and aquaponic‐cultivated *E*. *arvense* by evaluating (1) morphological characteristics, (2) active compounds using HPLC, (3) total phenolic and flavonoid contents, and (4) antioxidant activity (IC50, DPPH). No prior study has performed such a direct comparison for this species. This research examines these characteristics to identify the best cultivation method for achieving the highest quality and medicinal properties. Therefore, the novelty lies in the first comparative assessment of aquaponics versus wild conditions on this plant’s key bioactive traits. The findings can help better understand the impact of cultivation methods on the morphological, phytochemical, and antioxidant diversity of this plant and assist in developing its cultivation and breeding programs in the future.

## 2. Materials and Methods

### 2.1. Aquaponic System

The aquaponic system consisted of a 1000‐L fish tank stocked with 40 common carp fry (*Cyprinus carpio*; initial individual weight 50 g), which were fed daily with a commercial diet (Faradaneh Company, Iran) at 2% of their body weight. The system also included a mechanical filter tank (to remove solid particles and excess sediments), a biofilter tank (to remove gases such as methane and hydrogen sulfide), and a vertical raft plant cultivation tank. Water quality parameters were monitored weekly using Vaheb Company test kits (Iran) and maintained within optimal ranges for aquaponics throughout the cultivation period: pH = 6.0–7.0, dissolved oxygen > 5 mg/L (continuously monitored with an air pump and aeration stones), total ammonia < 1 mg/L, nitrite < 1 mg/L, nitrate < 150 mg/L, and water hardness within 100–300 mg/L as CaCO_3_. No chemical fertilizers were added; plant nutrients were supplied solely from fish waste. The aquaponic system was housed in a greenhouse with controlled environmental conditions: air temperature at 22°C–28°C, water temperature at 18°C–25°C (optimal compromise for fish, plants, and nitrifying bacteria), relative humidity at 55%–65%, and light provided by natural sunlight supplemented with LED‐grown lights (16/8 h light/dark photoperiod; photosynthetic photon flux density of 200–250 μmol·m^−2^·s^−1^ at the plant canopy level). The aquaponic system was established and allowed to cycle for 4 weeks prior to planting, and *E*. *arvense* rhizomes were then cultivated in the system for 12 weeks (from June to September 2023) before sample collection (Figure 1).

**FIGURE 1:**
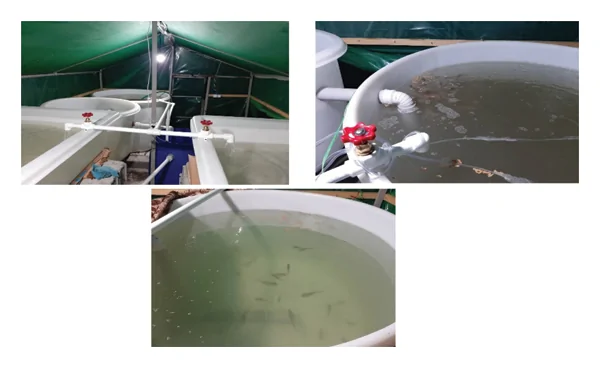
Aquaponic system.

### 2.2. Collection and Preparation of Plant Materials

In this study, wild *E*. *arvense* was collected from the Armudeh area in Ramsar city, Mazandaran province, Iran, in June 2023, at an altitude of 1785 m, with geographical coordinates N: 36.74185 and E: 50.55351. This information was recorded using a GPS device and registered under herbarium code 5799. The collected samples were verified by the Agricultural and Natural Resources Research and Education Center of Guilan Province, Iran. In this study, the rhizome of *E*. *arvense* was collected from its habitat in the Armudeh region and transferred to aquaponic systems (Figure 2).

**FIGURE 2:**
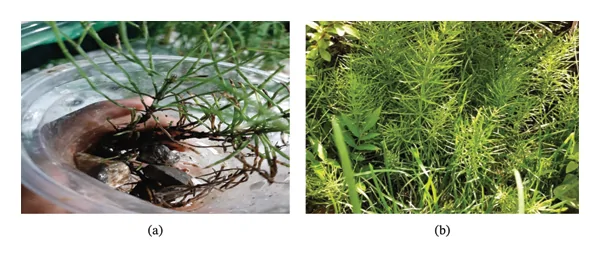
(a) *Equisetum arvense* rhizomes for aquaponics, (b) wild specimens.

### 2.3. Morphological Study of Wild *E*. *arvense* and Aquaponic Cultivation

Phenotypic traits, including height, internodal distance, stem diameter, number of leaves per node, number of leaflets per leaf, internodal distance per leaf, leaf color, and number of sheath teeth, were measured between wild and domesticated (cultivated in an aquaponic system) *E*. *arvense*. Measurements were taken using a ruler and the naked eye (Figure 3).

**FIGURE 3:**
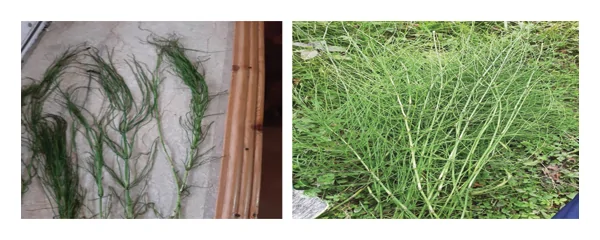
Measurement of morphological characteristics of *Equisetum arvense*.

### 2.4. Isolation of Extracts

The aerial parts of *E*. *arvense* were collected, cleaned, washed, and dried at room temperature (26°C–29°C) in the shade for 4–6 days. Then, the dried plant sample was powdered, and 50 g of the powder was extracted in 300 cc of hydroalcoholic solvent (methanol) using a Soxhlet apparatus for 4 h. After that, the resulting extract was dried in an oven at 40°C–50°C for 2–3 days. The dry powder of the obtained extract was mixed with dimethyl sulfoxide (DMSO) and tested after purification [14].

### 2.5. HPLC Assay

The powdered dried materials of *E*. *arvense* (1.5 g) were extracted three times with 20 mL of methanol at room temperature for 24 h each time. Eventually, the combined extracts were evaporated to dryness under reduced pressure using a Heidolph rotary vacuum evaporator. For chromatographic determination, 1 mg of the dried plant extract was dissolved in 1 mL of HPLC‐grade methanol, resulting in a final concentration of 1 mg/mL (1000 μg/mL). The solution was filtered through a 0.45‐μm syringe filter before injection. The validation of our analytical method followed the regulations of the International Council for Harmonization (ICH) for qualitative analysis. HPLC analysis was performed on a C18 reversed‐phase column (250 × 4.6 mm, 5 μm). The mobile phase consisted of solvent A: water containing 0.1% formic acid and solvent B: methanol (HPLC grade)**.** The flow rate was adjusted to 0.5 mL/min, and the injection volume was 20 μL. The gradient program was as follows: 0–15 min, 12% B; 15–60 min, 12%–22% B; 60–70 min, 22%–35% B; 70–80 min, 35%–60% B; 80–90 min, 60%–12% B. UV DAD was used to perform spectral scans over the range of 190–400 nm, and chromatograms were recorded at 280 nm for detection of phenolic compounds. The purpose of this HPLC analysis was primarily qualitative identification of compounds in the extracts. However, to enable comparative assessment between wild and aquaponically cultivated samples, the concentrations of identified compounds were estimated using a semi‐quantitative approach based on the relative peak area comparisons with single‐point external standards (100 μg/mL) under identical HPLC conditions. The reported values in Table 1 are therefore semi‐quantitative estimates for comparative purposes, not absolute quantitative concentrations. Complete validation parameters (calibration curves, LOD, and LOQ) were not calculated, as the primary objective of this study was to compare the presence/absence and relative abundance of phytochemical compounds between the two cultivation conditions. The repeatability of the method was assessed by three consecutive injections of the same sample, and the relative standard deviation (RSD) of retention times was < 2% [15].

**TABLE 1:** Semi‐quantitative estimation of compounds identified in wild and cultivated *Equisetum arvense* extracts based on HPLC peak area comparisons.

Active compounds	Number	
	Wild *Equisetum arvense* (mg/g)	Domesticated *Equisetum arvense* (mg/g)
1. Catechin	N.D	5.98
2. Chlorogenic acid	58	1.11
3. Caffeic acid	5.19	2.50
4. Vanillic acid	5.01	1.45
5. 2.5 DHB	N.D	1.53
6. p‐Coumaric acid	N.D	2.14
7. Ferulic acid	11.83	N.D
8. Rutin	10.98	2.62
9. Rosmarinic acid	5.86	7.34
10. Quercetin	1.53	N.D

*Note:* Values are approximate semi‐quantitative estimates (mg/g) based on relative peak area comparisons with external standards (single‐point calibration at 100 μg/mL). These values are intended for comparative assessment between the two cultivation systems, not as absolute quantitative concentrations.

Abbreviation: N.D = not detected.

### 2.6. Determination of TPC

Briefly, to each well of a 96‐well multiwell plate, 25 μL of the sample (1, 10, and 1000 μg/mL), 125 μL of Folin–Ciocalteu’s reagent (10% v/v in distilled water), and 100 μL of 7.5% (w/v) Na_2_CO_3_ were pipetted. During the oxidation of phenolic compounds, the phosphomolybdic and phosphotungstic acids contained in the Folin–Ciocalteu’s reagent were reduced to blue‐colored molybdenum and tungsten oxides. After one and a half hours, the absorbance of the blue coloration was measured at 765 nm against a blank sample. The measurements were compared to a standard curve of prepared gallic acid solutions (10, 50, 100, 250, 500, and 1000 mg L^−1^) and expressed as micrograms of gallic acid equivalents per mL of sample. All measurements were performed in three replicates [16].

### 2.7. Flavonoid Measurement

The determination of the total flavonoid content (TFC) was performed using the colorimetric aluminum chloride method. Briefly, 20 μL of the sample solution (1, 10, and 1000 μg/mL) were mixed with 80 μL of distilled water and 6 μL of NaNO_2_ (15% w/v) and then kept for 6 min. Afterward, 6 μL of 10% aluminum chloride, 80 μL of NaOH (4% w/v), and 8 μL of distilled water were pipetted into each well of a 96‐well multiwell plate, respectively. The mixture was then left at room temperature for 3 min. The absorbance of the reaction mixture was measured at 510 nm. TFCs were calculated as rutin equivalents from a calibration curve. The calibration curve was prepared by making rutin solutions at concentrations of 10, 50, 100, 250, 500, and 1000 mg L^−1^ in methanol. All measurements were performed in triplicate [17].

### 2.8. Antioxidant Capacity of DPPH Radicals

Stable 2,2‐diphenyl‐1‐picrylhydrazyl (DPPH) was used to determine the free radical scavenging activity of the extract. Two hundred microliters (200 μL) of the methanolic extract (various concentrations from 1000 μg to 5000 μg/mL) was added to 200 μL of a methanolic solution containing 0.5 mM DPPH. After 30 min at room temperature, the absorbance at 517 nm was recorded using the UV2100PC spectrophotometer. IC50 values, as the standard control, indicate the sample concentration required to scavenge 50% of DPPH free radicals. The DPPH assay was performed in triplicate [18].

### 2.9. Statistical Analysis

Morphological data were analyzed using an independent *t*‐test and presented as mean ± SD. Significance was set at *p* < 0.05 and *p* < 0.01. Superscript letters (a, b) and “ns” in Table 2 indicate significant and nonsignificant differences, respectively. All statistical analyses were performed using SPSS software (version 26.0; IBM Corp., Armonk, NY, USA).

**TABLE 2:** Morphological characteristics of wild and aquaponic‐cultivated plants at all growth stages.

Characteristics	Wild plant (mean ± SD)	Aquaponic plant (mean ± SD)	*p* value
Start Time	August 20–30	—	—
Plant Height (cm)	40.2 ± 2.8^a^	49.8 ± 3.1^b^	< 0.05
Stem Diameter (mm)	2.4 ± 0.3^a^	2.8 ± 0.2^b^	< 0.05
Node Spacing (cm)	2.4 ± 0.2^a^	3.2 ± 0.2^b^	< 0.01
Number of Leaves per Node	8.5 ± 1.2^a^	6.2 ± 1.5^b^	< 0.05
Number of Internodes per Leaf	13.5 ± 1.0^a^	14.0 ± 1.2^a^	ns
Internode Spacing per Leaf (mm)	4.5 ± 0.8^a^	6.0 ± 1.0^b^	< 0.05
Leaf Color	Dark Green	Dark Green	—
Number of Sheath teeth	3.5 ± 0.5^a^	3.5 ± 0.5^a^	ns

*Note:* Different superscript letters (a, b) in the same row indicate statistically significant differences (*p* < 0.05, independent *t*‐test).

Abbreviation: ns = not significant.

## 3. Result

### 3.1. Morphological Traits

Plant height was significantly greater in aquaponic plants (49.8 ± 3.1 cm) compared to that in wild plants (40.2 ± 2.8 cm; *p* < 0.05). Stem diameter was also significantly higher in aquaponic plants (2.8 ± 0.2 mm) than in wild plants (2.4 ± 0.3 mm; *p* < 0.05). Node spacing showed a highly significant difference (*p* < 0.01), with aquaponic plants having greater spacing (3.2 ± 0.2 cm) than wild plants (2.4 ± 0.2 cm). In contrast, the number of leaves per node was significantly higher in wild plants (8.5 ± 1.2) compared to that in aquaponic plants (6.2 ± 1.5; *p* < 0.05). The number of internodes per leaf showed no significant difference between wild (13.5 ± 1.0) and aquaponic plants (14.0 ± 1.2; ns). However, the internode spacing per leaf was significantly greater in aquaponic plants (6.0 ± 1.0 mm) than in wild plants (4.5 ± 0.8 mm; *p* < 0.05). The number of sheath teeth was similar between wild (3.5 ± 0.5) and aquaponic plants (3.5 ± 0.5; ns). No statistical comparison was performed for start time and leaf color due to the qualitative nature of these traits (Table 2).

### 3.2. Analysis of Active Compounds in *E*. *arvense* Extract

The semi‐quantitative analysis of active compounds in E. arvense extract, based on HPLC peak area comparisons (Figure 4), revealed significant differences between wild and domesticated samples. The values reported in Table 1 are semi‐quantitative estimates (mg/g) derived from HPLC chromatogram peak areas and should be interpreted as approximate indicators of relative abundance between the two cultivation systems, rather than absolute quantitative concentrations. In general, the wild sample had higher concentrations of chlorogenic acid (58 mg/g), ferulic acid (11.83 mg/g), and rutin (10.98 mg/g), indicating a greater potential for health benefits associated with these compounds. In contrast, the domesticated sample had a higher concentration of rosmarinic acid (7.34 mg/g). These findings suggest that wild cultivars may contain more beneficial compounds, while domesticated cultivars also have their own advantages (Table 1 and Figure 4).

**FIGURE 4:**
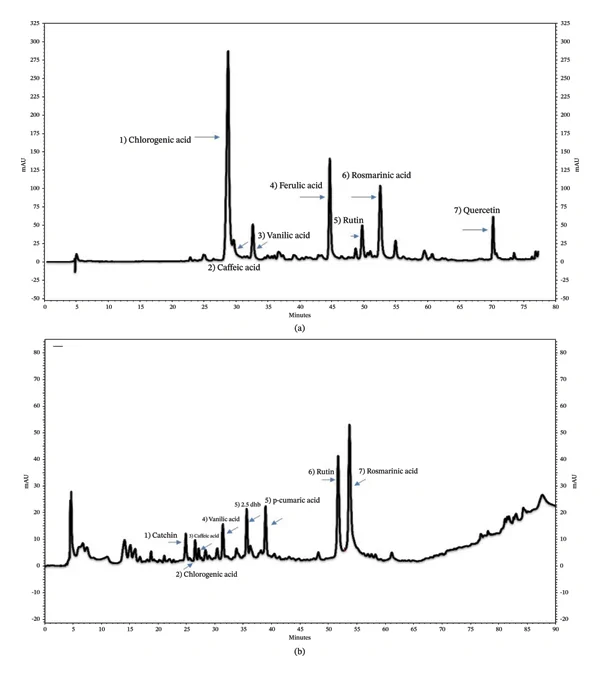
Chromatogram of plant extract *Equisetum arvense* ((a) wild, (b) domesticated).

### 3.3. Measurement of Total Phenol and Flavonoid Contents

The TPC of the methanolic extract of wild and domesticated *E*. *arvense* was 55.4 ± 0.6 and 45.4 ± 0.6 mg/g gallic acid equivalent (GAE), respectively. The TFC of wild and domesticated *E*. *arvense* was expressed as mg/g rutin equivalent. The TFC of the methanolic extract of wild and domesticated *E*. *arvense* was 31.25 ± 0.50 and 21.25 ± 0.50 mg rutin equivalent per gram of dry sample, respectively (Table 3).

**TABLE 3:** Total phenolic and flavonoid contents measured in the methanolic extract of *Equisetum arvense*.

Sample	Total phenol content (mg/g gallic acid equivalent (GAE))	Total flavonoid content (mg rutin equivalent per gram of dry sample)
Wild *E. arvense*	55.4 ± 0.6	31.25 ± 0.50
Domesticated *E. arvense*	45.4 ± 0.6	21.25 ± 0.50

### 3.4. Evaluation of Antioxidant Activity

The DPPH free radical scavenging rate and, consequently, the concentration of antioxidant compounds in wild and domesticated *E*. *arvense* plants (as shown in Table 4) were 282.83 and 182.83 μg/mL, respectively, compared to the IC50 value for BHT (butylhydroxytoluene), which was 8.3 μg/mL.

**TABLE 4:** Evaluation of antioxidant properties of *Equisetum arvense* plant.

Sample	Antioxidant activity (IC50: μg/mL)
Wild *E*. *arvense*	282.83
Domesticated *E*. *arvense*	182.83
BHT	8.3

## 4. Discussion

### 4.1. Morphological Evaluation of *E*. *arvense* Methanolic Extract

The morphological findings of this study indicate that aquaponic cultivation positively influences several growth parameters, particularly those related to vertical growth and structural strength, while wild plants show a trade‐off with greater leaf production per node. These observations are consistent with the statistical analyses (Table 2), where aquaponic plants had significantly greater height, stem diameter, node spacing, and internode spacing, whereas wild plants had significantly more leaves per node. No significant differences were observed between the two groups for number of internodes per leaf and number of sheath teeth. These findings align with recent reports showing that aquaponic systems improve morphological traits in various medicinal plants. In line with the results of this study, findings showed that the aquaponic cultivation system with mixed perlite and cocopeat substrates significantly improved the growth of basil cultivar “Purple” compared to that of “Green” and other substrates, confirming the positive effect of cultivation substrates on the morphological and physiological traits of the plant [19]. In another study, it was shown that the aquaponic system, by providing controlled and optimal conditions, helped improve the morphological characteristics of the Mentha plant, increasing height, stem diameter, and chlorophyll health, demonstrating high potential for optimizing the growth of this plant [20]. Additionally, Albadwawi et al. showed that the aquaponic system for the production of *Ocimum basilicum* L. under the conditions of the United Arab Emirates significantly increased morphological traits such as plant height, number of leaves, and stem diameter compared to traditional cultivation in a greenhouse. These findings indicate the high ability of the aquaponic system to enhance basil growth and development compared to soil cultivation methods [21]. Research results showed that water mint and peppermint in the aquaponic system performed best in terms of morphological traits, including leaf and shoot dry weight and leaf‐to‐stem ratio, confirming the high potential of these species for optimizing production in aquaponic systems [22].

### 4.2. Compounds Identified in *E*. *arvense* Extract

Analysis of active compounds in *E*. *arvense* extract shows significant differences between wild and domesticated samples. In general, the wild sample has a higher concentration of important compounds, with chlorogenic acid identified as the main compound, followed by ferulic acid and rutin as the major compounds in wild *E*. *arvense*. In the domesticated sample grown in aquaponics, rosmarinic acid is the highest, followed by catechin and rutin. To rule out analytical artifacts for compounds reported as not detected (N.D.) in one cultivation system, all samples were processed under identical HPLC conditions with repeatability (RSD < 2%). Therefore, the presence or absence of specific compounds (e.g., ferulic acid and quercetin in aquaponic samples; catechin and p‐coumaric acid in wild samples) is attributed to cultivation conditions rather than analytical or extraction variability. Wild plants face natural stresses that may upregulate certain phenolics, while the controlled aquaponic environment suppresses them. These findings are consistent with literature on secondary metabolite regulation. In this regard, the results of HPLC analysis from a study analyzing the ecotypes of *E*. *arvense* L. showed that five main phenolic acids were identified, including gallic acid, chlorogenic acid, p‐coumaric acid, isoquercitrin, and rutin. Among them, isoquercitrin was observed as the most abundant phenolic acid in the EA2 ecotype, which can be considered an important indicator in horsetail cultivation [9]. In another study, it was shown that the results of phytochemical analysis of *E*. *arvense* using the HPLC method indicate significant diversity in the phytochemical compounds of this plant. The concentrations of phenolic acids and flavonoids in different samples were significantly different, with the presence of compounds such as dicaffeic acid and various flavonoids serving as effective qualitative markers in evaluating the medicinal effects of this plant [23]. The results of another study showed that *E*. *arvense* contains numerous phenolic compounds and flavonoids, including phenolic acids such as caffeic and vanillic acids [15].

### 4.3. TPC and TFC

Comparative analysis of TPC and TFC between wild and aquaponically grown *E*. *arvense* revealed significantly higher levels of both phytochemicals in wild samples. This difference indicates that cultivation conditions strongly influence the accumulation of these secondary metabolites. The higher concentrations in wild plants likely reflect their exposure to natural environmental stresses, which are known to enhance phenolic and flavonoid biosynthesis as a defense response. In contrast, the controlled aquaponic environment with reduced stress may lead to lower accumulation of these compounds. Consistent with the results of the present study, the TPC was higher in wild *E*. *arvense* compared to that in aquaponically grown *E*. *arvense*. According to Peyghamzadeh et al., this diversity can be attributed to environmental stress factors that wild plants face, as phenolic compounds are one of the largest groups of plant phytochemicals produced in response to environmental challenges [24]. In this regard, research results showed that the TPC in horsetail reached its highest value of 1.744 mg GAE/g DW, while its flavonoid content was 1.062 mg RUT/g DW [25]. In a study that investigated the total phenol and flavonoid contents of horsetail in different ecotypes, the values were found to be 123.4 mg GAE/g DW and 45.30 mg RE/g DW, respectively [9]. Research results indicated that *E*. *arvense* L. is a rich source of phenolic compounds, with values of 82.63 mg GAE/g and a flavonoid content of 71.23 mg RE/g [15]. The controlled environment of the aquaponic system, while providing optimal growth conditions, may lead to reduced environmental stress and consequently a lower production of these defense compounds, as supported by Bukhari et al., who showed that culture conditions significantly affect the biosynthesis of secondary metabolites in *E. arvense* [26].

### 4.4. DPPH Free Radical Scavenging Activity

DPPH radical scavenging activity differed markedly between wild and aquaponically grown *E*. *arvense*. Aquaponic samples showed stronger antioxidant activity (lower IC50 values) than wild samples, with activity approaching that of the synthetic standard BHT. In contrast, wild plants exhibited comparatively lower radical scavenging capacity. These findings suggest that controlled aquaponic conditions may enhance antioxidant potential, likely due to cultivation‐induced differences in the accumulation of phenolic and flavonoid compounds. In a study evaluating the antioxidant capacity of *E*. *arvense* extracts using various methods such as the Cuprac test, DPPH method, and FRAP method, significant antioxidant activity was observed. The results indicated high concentrations of antioxidant compounds in the extracts, which can be considered an effective source for preventing oxidative damage. Additionally, comparisons with other medicinal plants showed that the antioxidant capacity of horsetail extracts is at a high level [15]. Dormousoglou et al. reported that all extracts of *E*. *arvense* L. contained significant quantities of phenolic and flavonoid compounds, with their antioxidant activity being markedly enhanced in DPPH, FRAP, and ABTS assays. Notably, the ethanol/water extract (S4) demonstrated the highest antioxidant activity. These observations suggest that extracts from this plant hold promise as effective antioxidant resources for pharmaceutical purposes [27]. In another study, the antioxidant activity of *E*. *arvense* L. extracts was measured using DPPH and hydroxyl radical scavenging assays. The n‐butanol extract showed the highest activity, with an EC50 of 0.65 mg/mL for DPPH and 0.74 mg/mL for hydroxyl radicals. Furthermore, the radical scavenging activity was significantly correlated with the TPC of the extracts [28]. The results of a study indicated the highest antioxidant activity in the hydroalcoholic extract of *E*. *arvense* L [29].

The superior antioxidant activity observed in aquaponic samples, despite their lower TPC/TFC compared to that in wild plants, can be explained by qualitative differences in their phenolic profiles and several underlying chemical mechanisms. The presence of rosmarinic acid as the dominant phenolic compound in aquaponic samples is particularly significant, as this compound possesses two catechol moieties that confer exceptional radical scavenging properties [30], whereas chlorogenic acid, which predominates in wild samples, has only one caffeoyl moiety [31]. This structural distinction is critical because rosmarinic acid benefits from intramolecular hydrogen bonding that stabilizes phenoxyl radicals and lowers bond dissociation energy, facilitating hydrogen atom transfer to free radicals [32]. Furthermore, rosmarinic acid exhibits superior metal‐chelating properties compared to chlorogenic acid, providing a dual antioxidant mechanism through both radical scavenging and metal chelation pathways [33]. The methodological distinction between the Folin–Ciocalteu and DPPH assays also warrants consideration; the Folin–Ciocalteu reagent primarily measures reducing capacity and may react nonspecifically with other reducing substances, whereas the DPPH assay specifically evaluates radical scavenging activity. The controlled aquaponic environment may promote the biosynthesis of specific phenolic compounds with higher antioxidant efficacy, and synergistic interactions among rosmarinic acid, catechin, and rutin may enhance the overall antioxidant activity beyond the sum of individual compound effects. These mechanistic insights confirm that TPC alone is an insufficient predictor of antioxidant efficacy, and the specific chemical profile of phenolic compounds must be considered when evaluating therapeutic potential.

The results of this study showed that aquaponic systems can increase certain growth parameters, such as height and stem diameter, which may lead to differences in leaf growth and node spacing. Understanding these morphological traits is crucial for optimizing cultivation practices and maximizing the potential of *E*. *arvense* as a valuable plant for various applications, including its use in herbal medicine and as a source of phytochemicals. On the other hand, the phytochemical compounds present in *E*. *arvense* plant extracts are affected by different cultivation methods, particularly wild and aquaponic cultivation. Wild plants produce higher concentrations of phenolic compounds and flavonoids, including chlorogenic and ferulic acids, due to exposure to environmental stresses. These compounds act as defense mechanisms and are associated with antioxidant properties. Interestingly, aquaponically cultivated plants, despite their lower phenolic content, exhibited superior antioxidant activity, a finding likely attributed to the controlled conditions that promote the accumulation of specific bioactive compounds, such as rosmarinic acid. This metabolite, which is favored in aquaponic systems, possesses considerable antioxidant and anti‐inflammatory properties. These changes in chemical composition and biological activities can have profound effects on the medicinal properties of the plant and enhance our understanding of its applications in traditional and modern medicine.

### 4.5. Limitations of the Study

Several limitations of this study should be acknowledged. Wild samples were collected from a single geographical location (Armudeh region, Ramsar, Iran), which limits generalizability to other regions. The aquaponic system was evaluated under specific controlled conditions, and variations in parameters could affect outcomes. The cultivation period was limited, leaving long‐term effects on phytochemical profiles unknown. No statistical comparison was performed for individual HPLC compounds; therefore, the observed differences are descriptive rather than inferential. Furthermore, the HPLC analysis was performed using a semi‐quantitative approach with single‐point external standards; therefore, the values in Table 1 should be interpreted as estimates of relative abundance for comparative purposes. The observed differences are descriptive rather than inferential. Additionally, antioxidant activity was assessed using only the DPPH assay. Due to equipment limitations, lack of access to specific assay kits (e.g., SOD, CAT, APX), and time constraints, these analyses were not performed and are recommended for future research. Future studies should include multiple wild populations, optimize aquaponic parameters, extend cultivation periods, and employ additional bioactivity assays.

## 5. Conclusion

Finally, this study reveals that cultivation method significantly influences both the phytochemical profile and bioactivity of *E*. *arvense*. Although the HPLC data in this study are semi‐quantitative and intended for comparative purposes between the two cultivation systems, they clearly demonstrate that cultivation method significantly influences the phytochemical profile of *E. arvense*. Wild plants excel in the diversity and concentration of phenolics and flavonoids, whereas aquaponically cultivated plants exhibit stronger antioxidant activity despite their lower phenolic content. These findings suggest that each cultivation method offers distinct advantages, and neither is absolutely superior. However, aquaponic cultivation can be considered a sustainable and efficient method for the production of medicinal plants by increasing growth parameters. The choice of cultivation method should therefore be guided by the specific medicinal application and desired bioactive properties. The findings of this research can help optimize cultivation methods and enhance the medicinal potential of plants in the future, pointing to the development of effective strategies for the production of these plants under controlled conditions. This study emphasizes the importance of understanding the effects of cultivation methods on active compounds, particularly phenolics and flavonoids, in order to improve their quality and therapeutic efficacy in medicinal applications.

## Funding

No funding was received for this manuscript.

## Conflicts of Interest

The authors declare no conflicts of interest.

## Data Availability

The data that support the findings of this study are available from the corresponding author upon reasonable request.
